# WASH activation controls endosomal recycling and EGFR and Hippo signaling during tumor-suppressive cell competition

**DOI:** 10.1038/s41467-022-34067-1

**Published:** 2022-10-21

**Authors:** Dan Liu, Vasilios Tsarouhas, Christos Samakovlis

**Affiliations:** 1 Science for Life Laboratory, Department of Molecular Biosciences, The Wenner-Gren Institute, Stockholm University, SE-10691 Stockholm, Sweden; 2 Cardiopulmonary Institute, Justus Liebig University of Giessen, Giessen, Germany

**Keywords:** Growth factor signalling, Retromer

## Abstract

Cell competition is a conserved homeostatic mechanism whereby epithelial cells eliminate neighbors with lower fitness. Cell communication at the interface of wild-type “winner” cells and polarity-deficient (*scrib*^−/−^) “losers” is established through Sas-mediated Ptp10D activation in polarity-deficient cells. This tumor-suppressive cell competition restrains EGFR and Hippo signaling and enables Eiger-JNK mediated apoptosis in *scrib*^−/−^ clones. Here, we show that the activation state of the endosomal actin regulator WASH is a central node linking EGFR and Hippo signaling activation. The tyrosine kinase Btk29A and its substrate WASH are required downstream of Ptp10D for “loser” cell elimination. Constitutively active, phosphomimetic WASH is sufficient to induce both EGFR and Yki activation leading to overgrowth. On the mechanistic level we show that Ptp10D is recycled by the WASH/retromer complex, while EGFR is recycled by the WASH/retriever complex. Constitutive WASH activation selectively interferes with retromer function leading to Ptp10D mistargeting while promoting EGFR recycling and signaling activation. Phospho-WASH also activates aberrant Arp2/3 actin polymerization, leading to cytoskeletal imbalance, Yki activation and reduced apoptosis. Selective manipulation of WASH phosphorylation on sorting endosomes may restrict epithelial tumorous growth.

## Abstract (web summary)

Polarity-deficient epithelial cells are eliminated through Sas-Ptp10D-mediated suppression of EGFR signalling during cell competition. Here the authors show the constitutively active, phosphomimetic WASH can activate both EGFR and Hippo signalling and thereby, induces the overgrowth of these polarity-deficient cells in *Drosophila* and in proliferating epithelial cells, WASH activation differentially affects the separate recycling routes of EGFR and its inhibitor Ptp10D.

## Introduction

Cell competition during epithelial development is a common mechanism operating in *Drosophila* and mammals^1,2^. To maintain tissue homeostasis, epithelial cells communicate with their neighbors and remove unfit or mutant cells with oncogenic mutations. Cell competition can be induced by competing for survival factors and nutrients^3–11^, fitness sensing^12–15^ or mechanical force^16^. For instance, mutations in the tumor suppressor gene *scribble* (*scrib*), involved in epithelial polarization and endocytosis, generate imaginal disc overgrowth. However, when surrounded by wild-type neighbors, *scrib*^−/−^ cells are eliminated through Eiger activation of JNK-mediated apoptosis^17–19^. In eye-antennal imaginal discs, elimination initiates from translocation of the receptor tyrosine phosphatase Ptp10D to the lateral membranes of *scrib*^−/−^ cells, where it associates with its ligand Sas at the adjacent membranes of wild-type cells. This enables Ptp10D to suppress EGFR signaling in *scrib*^−/−^ leading to their elimination through apoptosis^14^. The underlying mechanism of how interface communication between tumor and normal cells is maintained and how it controls signaling in *scrib*^−/−^ clones remains unclear. WASH (Wiskott–Aldrich syndrome protein and SCAR homologue), controls endosomal fission and cargo sorting by facilitating F-actin polymerization via the Arp2/3 complex^20–22^. Mammalian and *Drosophila* WASH associate with SWIP, Strumpellin, FAM21 and CCDC53 to form the WASH regulatory complex (SHRC)^23–25^. WASH is required for retromer function in mammalian cell lines^20,21^ and for selective retrograde recycling in *Drosophila*^26^. In the *Drosophila* embryo, the Ptp10D/Btk29A/WASH circuit controls the initiation of an apical endocytosis burst leading to luminal protein clearance and airway maturation. Btk29A phosphorylates and activates WASH, while the receptor tyrosine phosphatase Ptp10D antagonizes WASH phosphorylation. This mode of WASH activation by phosphorylation also operates in mouse fibroblasts^27^.

In this work, we show that WASH functions downstream of Ptp10D/Btk29A in the *scrib*^−/−^-induced tumor-suppressive cell competition. A phosphomimetic WASH construct is sufficient to induce over-proliferation in *scrib*^−/−^ clones by activating EGFR signaling and the Hippo effector yki. Our work proposes a bifurcate recycling mechanism for EGFR and its inhibitor Ptp10D in epithelial cells during tumor-suppressive cell competition. Phospho-WASH is differentially regulated in the distinct recycling routes, offering possibilities to restrain aberrant growth signaling from EGFR.

## Results

### WASH functions downstream of Ptp10D/Btk29A in *Scrib*^−/−^-induced tumor-suppressive cell competition model

**Fig. 1:**
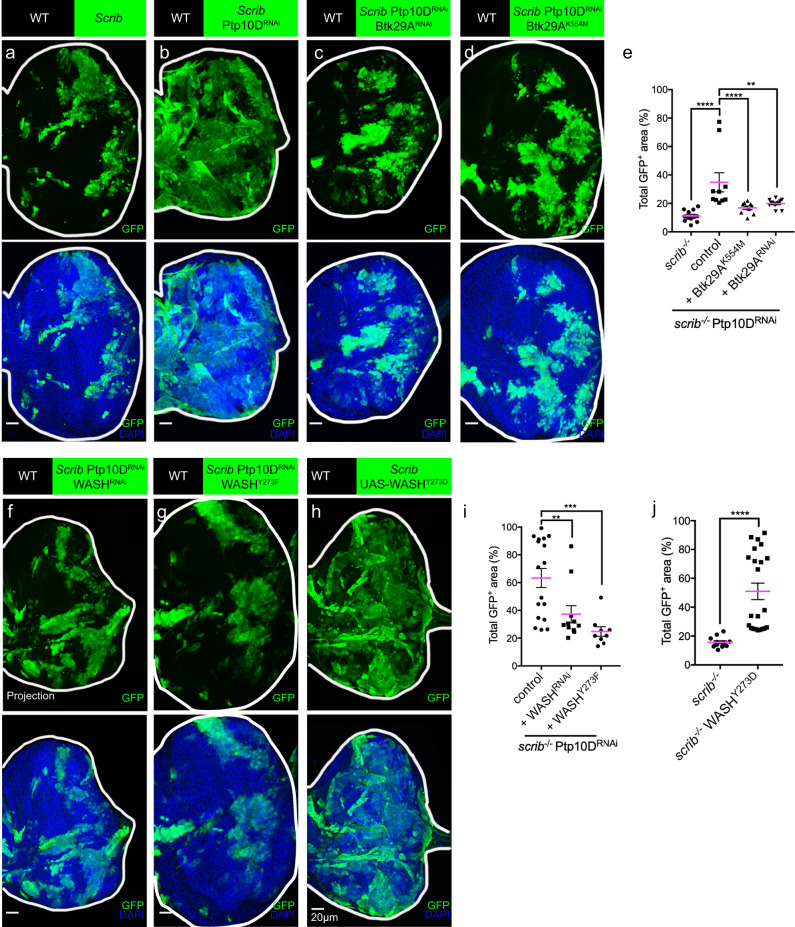
Ptp10D/Btk29A/WASH circuit acts in *scrib*^−/−^-induced tumor-suppressive cell competition model. **a–d** Eye-discs bearing MARCM-induced mosaics of GFP-labeled *scrib*^−/−^ (**a**), *scrib*^−/−^ Ptp10D^RNAi^ (**b**), *scrib*^−/−^Ptp10D^RNAi^, Btk29A^RNAi^ (**c**), and *scrib*^−/−^Ptp10D^RNAi^, Btk29A^K554M^ (**d**) clones immunostained with anti-GFP (green) and DAPI (blue). Scale bars, 20 μm (**e**) ﻿Quantification for total GFP-positive (GFP^+^) area (%) of the eye-discs bearing GFP-labelled *scrib*^−/−^ (*n* = 12, number of eye discs), *scrib*^−/−^ Ptp10D^RNAi^ (*n* = 10), *scrib*^−/−^Ptp10D^RNAi^ Btk29A^K554M^ (*n* = 10), *scrib*^−/−^ Ptp10D^RNAi^ Btk29A^RNAi^ (*n* = 13). Data are mean ± s.e.m; **P < 0.005, ****P < 0.0001, by two tailed unpaired Mann-Whitney U-test. **f–h** Eye-discs bearing MARCM-induced mosaics of GFP-labeled *scrib*^−/−^Ptp10D^RNAi^, WASH^RNAi^ (**f**) *scrib*^−/−^Ptp10D^RNAi^,WASH^Y273F^ (**g**), and *scrib*^−/−^ WASH^Y273D^ (**h**) clones immunostained with anti-GFP (green) and DAPI (blue). (**i**) Quantification of total GFP-positive (GFP^+^) area (%) of *scrib*^−/−^ Ptp10D^RNAi^ (*n* = 17, number of eye discs), *scrib*^−/−^Ptp10D^RNAi^, WASH^RNAi^ (*n* = 10), *scrib*^−/−^Ptp10D^RNAi^, WASH^Y273F^ *(n* = 9). Scale bars, 20 μm. Data are mean ± s.e.m; **P < 0.005, ***P < 0.0005 by two tailed unpaired Mann-Whitney U-test. **j** Quantification of total GFP-positive (GFP^+^) area (%) of *scrib*^−/−^ (*n* = 12) and *scrib*^−/−^WASH^Y273D^ (*n* = 22). Data are mean ± s.e.m^;^ ****P < 0.0001 by Mann-Whitney U-test.

*scrib* mutant clones are often eliminated from the eye disc epithelium, when surrounded by wild-type cells whereas *scrib*^−/−^ Ptp10D^RNAi^ clones in the same settings show overgrowth^14^ (Fig. 1a, b, e). Since Ptp10D levels become reduced in the posterior compartment in wing-disc of *enGal4* > UAS-GFP, Ptp10D-RNAi, we conclude that the RNAi construct knocks down *Ptp10D* function efficiently (Supplementary Fig 1m). Ptp10D antagonizes Btk29A and Btk29A phosphorylates and activates WASH during airway maturation^27^, we thus explored the role of Ptp10D/Btk29A/WASH circuit in the *scrib*^−/−^ cell clones. First, we decreased the activity of Btk29A by using Btk29A^RNAi^ or by overexpressing a kinase-dead form of Btk29A (Btk29A^KD^ or Btk29A^K554M^) in the *scrib*^−/−^ Ptp10D^RNAi^ clones. Dampening Btk29A activity strongly suppressed *scrib*^−/−^ Ptp10D^RNAi^ clone overgrowth, suggesting that Btk29A antagonizes Ptp10D in this tumor-suppressive cell competition model (Fig. 1b–e). WASH depletion by RNAi dramatically decreased the size of *scrib*^−/−^ clones co-expressing Ptp10D^RNAi^ (Fig. 1f, i), indicating that WASH acts downstream of Ptp10D also in the *scrib*^−/−^ cells. WASH levels are dramatically reduced in the posterior compartment in wing-disc of *enGal4* > UAS-GFP,UAS-WASH-RNAi showing the efficiency of WASH-RNAi (Supplementary Fig. 1n). WASH can be activated upon phosphorylation of its conserved tyrosine 273 by Btk29A^27^. Thus, we overexpressed the phosphomimetic form, WASH^Y273D^ (phospho-WASH^27^) in the *scrib* mutant clones. The *scrib*^−/−^ WASH^Y273D^ clones strongly resembled the overgrowth phenotype of *scrib*^−/−^ Ptp10D^RNAi^ clones (Fig. 1h, j), and similarly to *scrib*^−/−^ Ptp10D^RNAi^ clones, contained more Phospho-Histone 3 (PH3) marked dividing cells than *scrib*^−/−^ clones (Supplementary Fig. 1a–d). The wild-type form of WASH (WASH^WT^) overexpression also induced overgrowth of *scrib*^−/−^ clones but the growth phenotype appeared milder compared to the *scrib*^−/−^ WASH^Y273D^ clones (Supplementary Fig. 1o). This may suggest that the activity of the endogenous tyrosine kinase Btk29A is constitutively limited. To further test the role of Y273 phosphorylation in WASH in imaginal discs, we used a wing pouch-specific driver *Nubbin-Gal4* to overexpress different WASH variants and analyzed wing sizes of adult female flies. Overexpression of phosphomimetic WASH^Y273D^ but not WASH^WT^ showed increased wing sizes compared to control flies (*Nub-Gal4* > CD8-GFP). On the other hand, overexpression of a non-phosphorylatable form, WASH^Y273F27^ or *WASH* knockdown by RNAi showed reduced wing sizes (Supplementary Fig. 1p). In a complementary experiment, the growth of *scrib*^−/−^ Ptp10D^RNAi^ clones could be suppressed by overexpressing a non-phophorylatable WASH^Y273F^ (Fig. 1g, i). Our genetic experiments suggest that phosphorylation of a single tyrosine 273 in WASH can efficiently induce cell proliferation and tumor growth in the *scrib*^−/−^ clones. To further investigate how WASH^Y273D^ overexpression induces growth in *scrib* mutant clones, we examined the levels of *Drosophila* cell death effector caspases Dcp1 and DrICE^28–30^. The numbers of Dcp1-positive, dying cells were reduced both in the perimeter area and in the center of *scrib*^−/−^ Ptp10D^RNAi^ and *scrib*^−/−^ WASH^Y273D^ clones, compared to *scrib*^−/−^ clone cells (Supplementary Fig. 1e, f). Similarly, the labelling with an antibody against the activated form of DrICE showed much-reduced staining in *scrib*^−/−^ Ptp10D^RNAi^ and *scrib*^−/−^ WASH^Y273D^ clones, compared to *scrib*^−/−^ clones (Supplementary Fig. 1j–l). This suggests that the overgrowth phenotypes of the *scrib*^−/−^ Ptp10D^RNAi^ and *scrib*^−/−^ WASH^Y273D^ clones at least partly due to apoptosis inhibition. However, we detected similar MMP1 expression in *scrib*^−/−^, *scrib*^−/−^ Ptp10D^RNAi^ and *scrib*^−/−^WASH^Y273D^ clones, suggesting that the JNK pathway is stimulated in both elimination-fated “loser cells” and overgrowth-fated “winners” (Supplementary Fig. 1g–l). These data suggest that constitutive WASH phosphorylation is sufficient to suppress JNK-dependent apoptosis in *scrib*^−/−^ clones. Altogether, the data support a role of Ptp10D/Btk29A/WASH circuit in the *scrib*^−/−^ model of tumor-suppressive cell competition.

### WASH^Y273D^ overexpression induces punctuated F-actin and activates yki in the *scrib* mutant clones

**Fig. 2:**
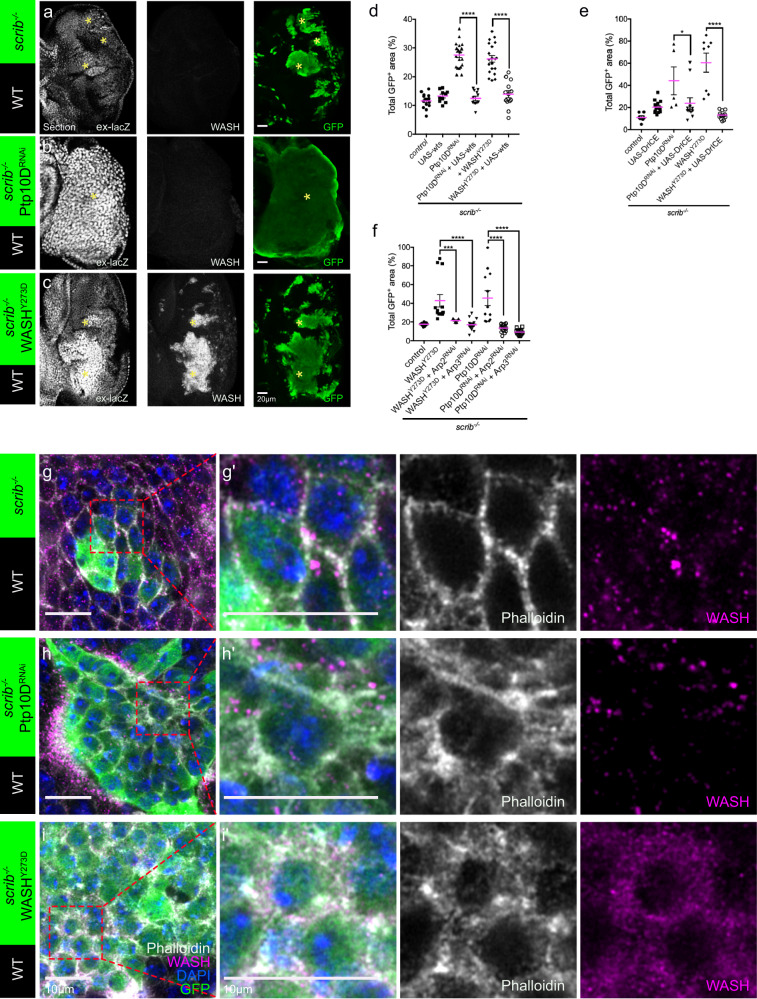
Phosphomimetic WASH^Y273D^ overexpression induces Arp2/3 dependent F-actin polymerization and yki activation in *scrib* mutant clones. (**a**–**c**) Eye-discs of ex-lacZ/+ fly bearing GFP-labelled *scrib*^−/−^ (**a**), *scrib*^−/−^ Ptp10D^RNAi^ (**b**) or *scrib*^−/−^ WASH^Y273D^ clones (**c**) immunostained with anti-GFP (green), anti-ß-gal (gray), anti-WASH (gray) and DAPI (blue). Scale bars, 20 μm. The yellow stars marked the GFP-labelled clones. (**d**) Quantification of total GFP^+^ area (%) of GFP-labeled clones of *scrib*^−/−^ (*n* = 15, number of eye discs), *scrib*^−/−^ UAS-wts (*n* = 10), *scrib*^−/−^ Ptp10D^RNAi^ (*n* = 21), *scrib*^−/−^ Ptp10D^RNAi^, UAS-wts (*n* = 12), *scrib*^−/−^ WASH^Y273D^ (*n* = 20) and *scrib*^−/−^ WASH^Y273D^, UAS-wts (*n* = 13). Data are mean ± s.e.m; ****P < 0.0001 by two tailed unpaired Mann-Whitney U-test. (**e**) Quantification of total GFP^+^ area (%) in genotypes shown in *scrib*^−/−^ (*n* = 7, number of eye discs), *scrib*^−/−^ UAS-DrICE (*n* = 14), *scrib*^−/−^ Ptp10D^RNAi^ (*n* = 5), *scrib*^−/−^ Ptp10D^RNAi^ UAS-DrICE (*n* = 11), *scrib*^−/−^ WASH^Y273D^ *(n* = 8), *scrib*^−/−^ WASH^Y273D^ UAS-DrICE (*n* = 12). Data are mean ± s.e.m; *P < 0.05, ****P < 0.0001 by Mann-Whitney U-test. (f) Quantification for total GFP^+^ area (%) of GFP-labeled *scrib*^−/−^(*n* = 9), *scrib*^−/−^ WASH^Y273D^ (n ^*=*^ 13, number of eye discs), *scrib*^−/−^ WASH^Y273D^, Arp2^RNAi^ (*n* = 7), *scrib*^−/−^ WASH^Y273D^, Arp3^RNAi^ (*n* = 16), *scrib*^−/−^ Ptp10D^RNAi^ (*n* = 12), *scrib*^−/−^ Ptp10D^RNAi^, Arp2^RNAi^ (*n* = 16) and *scrib*^−/−^ Ptp10D^RNAi^, Arp3^RNAi^ (*n* = 16) clones. Data are mean ± s.e.m; ***P < 0.0005, ****P < 0.0001 by two tailed unpaired Mann-Whitney U-test. (**g**–**i**) Eye-discs bearing GFP-labelled *scrib*^−/−^ (**g**), *scrib*^−/−^ Ptp10D^RNAi^ (**h**), and *scrib*^−/−^ WASH^Y273D^ (**i**) clones immunostained with phalloidin (gray), anti-GFP (green), anti-WASH (magenta) and DAPI (blue). (**g**’) (**h**’) (**i**’) show magnified images of (**g**) (**h**) (**i**). Scale bars, 10 μm.

Hippo pathway activation in polarity-deficient cells underlies the overgrowth of *scrib*^−/−^ Ptp10D^RNAi^ clones^14,31^. In imaginal discs, the transcriptional co-activator, yorkie (yki) promotes growth and inhibits apoptosis by activating proliferative and anti-apoptotic genes, such as *expanded* (*ex*) and *Drosophila* inhibitor of apoptosis-1 (*Diap1*). Hippo signaling controls growth by activating warts (wts), a kinase that directly phosphorylates and inhibits yki activity^32–34^. We examined the expression of *ex-lacZ*, a yki transcriptional reporter in the tumor-suppressive cell competition model^35^. Both the *scrib*^−/−^Ptp10D^RNAi^ and *scrib*^−/−^ WASH^Y273D^ clones showed elevated *ex-lacZ* levels compared to *scrib*^−/−^ (Fig. 2a–c). Furthermore, immunostaining of anti-DIAP1 showed increased DIAP1 in *scrib*^−/−^Ptp10D^RNAi^ and *scrib*^−/−^ WASH^Y273D^ clones compared to *scrib*^−/−^ (Supplementary Fig. 2b–d’). These results suggest that yki is activated by WASH^Y273D^. Consistent with these results, overexpression of UAS-Wts strongly suppressed the overgrowth phenotype of both *scrib*^−/−^Ptp10D^RNAi^ and *scrib*^−/−^ WASH^Y273D^ “winner” clones (Fig. 2d). Further, the size of *scrib*^−/−^ Ptp10D^RNAi^ and *scrib*^−/−^ WASH^Y273D^ clones was significantly reduced upon overexpression of DrICE^29,36,37^ (an effector caspase, which is inhibited by the yki transcriptional target *Diap1* (Fig. 2e). These data suggest that yki activation is required for apoptosis inhibition and overgrowth of both *scrib*^−/−^ WASH^Y273D^ and *scrib*^−/−^ Ptp10D^RNAi^. Yki can be directly stimulated by tensions mediated by imbalances in the cortical F-actin cytoskeleton^35,38,39^. The characteristic pattern of cortical phalloidin staining in *scrib*^−/−^ cells was severely disrupted in *scrib*^−/−^ Ptp10D^RNAi^ clones, where F-actin was increased inside the cells^14^ (Fig. 2g, h’). In *scrib*^−/−^ WASH^Y273D^ clones, the F-actin staining was intracellular and more punctate, resembling the localization of overexpressed WASH^Y273D^ and the cortical F-actin was disrupted (Fig. 2i-i’). Consistently, similar F-actin structures were detected in the GFP-labeled posterior compartment (P-compartment) of wing-discs with *engrailed-GAL4::UAS-GFP* (*enGAL4* > GFP) driven WASH^Y273D^ overexpression (Supplementary Fig. 2a-a’). WASH facilitates F-actin polymerization via the Arp2/3 complex to regulate endosome scission via the Arp2/3 complex^20,21^. Therefore, we tested whether the *scrib*^−/−^ WASH^Y273D^ tumorous phenotype also rely on the Arp2/3 complex. Indeed, depletion of either Arp2 or Arp3 using RNAi strongly suppressed the overgrowth in *scrib*^−/−^ clones induced by Ptp10D^RNAi^ or WASH^Y273D^ (Fig. 2f), indicating that tumorous growth requires Arp2/3 induced F-actin polymerization, presumably on endosomes. Further, the lowered DIAP1 levels when co-expressing Arp3^RNAi^ suggest that yki activation rely on Arp3 in *scrib*^−/−^ Ptp10D^RNAi^ or *scrib*^−/−^ WASH^Y273D^ clones (Supplementary Fig. 2b–f’). Altogether, these data argue that the endosomal activity of phospho-WASH is tightly regulated to balance endosomal versus cortical actin polymerization and yki activation.

### Phospho-WASH regulates endocytic trafficking of Ptp10D and EGFR to control EGFR signaling and aberrant growth

**Fig. 3:**
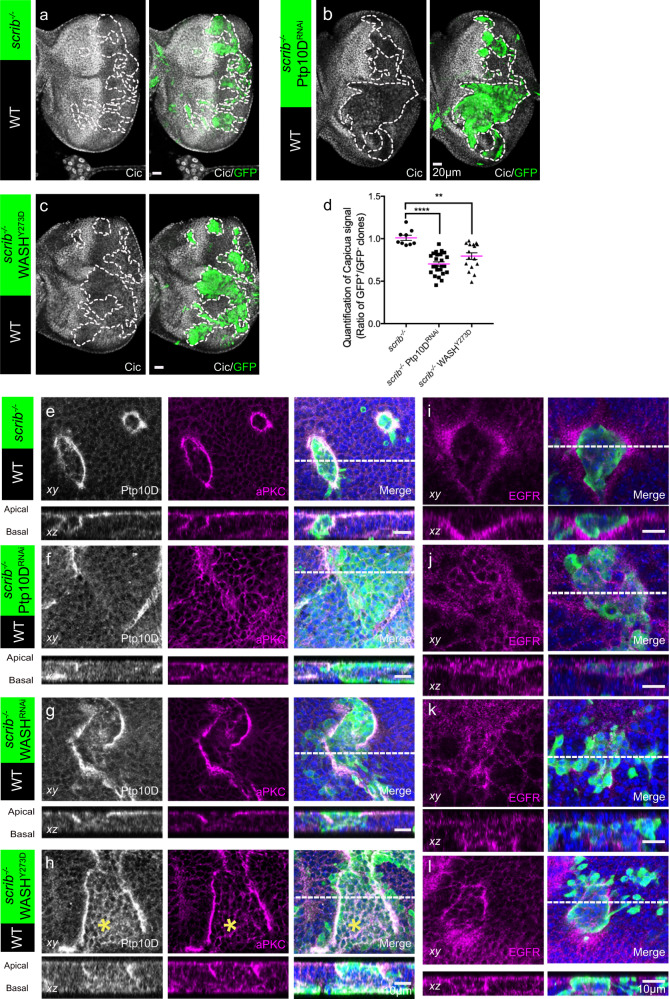
Phospho-WASH is sufficient to activate EGFR signaling in *scrib* mutant clones through regulating trafficking of Ptp10D and EGFR. **a**–**c** Eye-discs bearing GFP-labelled *scrib*^−/−^(**a**), *scrib*^−/−^ Ptp10D^RNAi^ (**b**), *scrib*^−/−^ WASH^Y273D^ (**c**) clones immunostained with anti-Cic (gray) and anti-GFP (green). Scale bars, 20 μm. **d** Quantification for Capicua signal (GFP-positive / negative clones ratio) in the *scrib*^−/−^ (*n* = 9, number of clones), *scrib*^−/−^ Ptp10D^RNAi^ (*n* = 24) and *scrib*^−/−^ WASH^Y273D^ (*n* = 16) clones. Data are mean ± s.e.m; **P < 0.005, ****P < 0.0001 by two-tailed unpaired Mann-Whitney U-test. **e–h** Immunochemistry analysis for Ptp10D and aPKC. Top images show xy confocal sections of eye disc bearing GFP-labelled *scrib*^−/−^ (**e**), *scrib*^−/−^ Ptp10D^RNAi^ (**f**), *scrib*^−/−^ WASH^RNAi^ (**g**), *scrib*^−/−^ WASH^Y273D^ (**h**) clones immunostained with anti-Ptp10D (gray), anti-aPKC (magenta), anti-GFP (green) and DAPI (blue); bottom images show xz cross sections. Dashed lines in the top right images with all the channels mark the positions of the cross-sections in the bottom images. Scale bars, 10 μm. **i–l** Immunochemistry analysis for EGFR. Top images show xy confocal sections of eye disc bearing GFP-labelled *scrib*^−/−^ (**i**), *scrib*^−/−^ Ptp10D^RNAi^ (**j**), *scrib*^−/−^ WASH^RNAi^ (**k**), and *scrib*^−/−^ WASH^Y273D^ (**l**) clones immunostained with anti-EGFR (magenta), anti-GFP (green) and DAPI (blue); bottom images show xz cross sections. Dashed lines in the top right images with all the channels mark the positions of the cross-sections in the bottom images. Scale bars, 10 μm.

Ptp10D is a negative regulator of EGFR signaling^40^ and elevated EGFR signaling can change the “loser cell” fate of *scrib* mutant cells to “winners”^35^. To assess EGFR signaling, we stained *scrib*^−/−^ WASH^Y273D^ and *scrib*^−/−^ Ptp10D^RNAi^ clones for Capicua (Cic), a transcriptional repressor downstream of EGFR pathway. Since Cic downregulation represents an upregulation of EGFR signaling^41^, we quantified the ratio of Cic signal in GFP positive and GFP negative cells. We detected similarly decreased Cic and upregulated EGFR signaling in *scrib*^−/−^ WASH^Y273D^ and *scrib*^−/−^ Ptp10D^RNAi^ clones (Fig. 3a–d). Thus, active WASH induces *scrib*^−/−^ overgrowth through upregulating EGFR signaling. This was further supported by the size reduction of *scrib*^−/−^ WASH^Y273D^ and *scrib*^−/−^ Ptp10D^RNAi^ clones expressing a dominant negative form of EGFR (EGFR^DN^) (Supplementary Fig. 3a–e). We first considered whether WASH may interfere with EGFR signaling downstream or parallel of Ras, a key GTPase in EGFR pathway activation. Hence, we generated mosaic clones using UAS-Ras^V12^ a constitutively active form of Ras. Ras^V12^ overexpression alone induces growth of eye imaginal disc and *scrib*^−/−^ Ras^V12^ clones develop into malignant tumors^42^. However, neither WASH^Y273D^ overexpression nor WASH^RNAi^ inactivation altered the aberrant overgrowth and Cic level changes driven by overexpression of Ras^V12^ in a wild-type background or in *scrib*^−/−^ clones (Supplementary Fig. 3f–i). These data argue against potential WASH functions in parallel or downstream of Ras. Because the Arp2/3 components were crucial for WASH function during tumor-suppressive cell competition, we examined whether WASH may impact on EGFR signaling through regulating endosomal actin polymerization. We first analyzed the localization of Ptp10D, aPKC and EGFR by immunofluorescence. All three proteins relocalized to the boundary of wild-type and *scrib*^−/−^ clones (Fig. 3e, i). In *scrib*^−/−^ Ptp10D^RNAi^ clones, Ptp10D was reduced as expected, but aPKC and EGFR were surprisingly increased inside the clones (Fig. 3f, j). Upon depletion of WASH in *scrib*^−/−^ clones, the relocalization of Ptp10D, aPKC and EGFR was impaired and the three proteins appeared cytoplasmic in the clones, indicating that the interface localization of Ptp10D/aPKC and EGFR requires WASH (Fig. 3g, k). Notably, WASH^Y273D^ overexpression in the *scrib*^−/−^ clones generated defects in Ptp10D interface localization and drastically increased Ptp10D and aPKC levels (Fig. 3h). Nonetheless, EGFR localization was unaffected in the interface membrane of *scrib*^−/−^ clones expressing WASH^Y273D^ (Fig. 3l). These data argue that constitutively active WASH selectively interferes with Ptp10D and aPKC localization. Moreover, Ptp10D/aPKC and EGFR were disrupted in the interface of *scrib*^−/−^ WASH^Y273F^ clones and their intensities were slightly increased within the clones, suggesting the non-phosphorylatable WASH^Y273F^ may interrupt the function of endogenous WASH (Supplementary Fig. 3j, k). Collectively, our results suggest that phospho-WASH controls EGFR signaling upstream of Ras, possibly through trafficking EGFR and its negative regulator Ptp10D.

### Retromer and retriever/CCC complexes maintain membrane localization of Ptp10D and EGFR, respectively

Previous work showed that WASH recycles internalized cargos either with the retromer or the retriever/CCC complexes^21,26,43–46^.The diverse effects of phospho-WASH in Ptp10D, aPKC and EGFR localization suggest that Ptp10D and EGFR may follow distinct WASH-dependent, recycling routes to the clone interface. To test this hypothesis, we firstly analyzed the localization of Ptp10D, aPKC and EGFR by knocking-down retromer components in wing discs with *enGAL4* > GFP. Downregulation of the retromer subunits Vps26, Vps35, Vps29 or a retromer-specific sorting nexin SNX27 in the P-compartment (expressing GFP) showed defective apical localization of Ptp10D and punctuated Ptp10D, compared to cells in the control A-compartment (Supplementary Fig. 4a–f). Thus, we conclude that the retromer complex controls the apical Ptp10D localization in epithelial cells. Surprisingly, apical localization of EGFR was only affected by Vps29^RNAi^ but not Vps35^RNAi^, Vps26^RNAi^ or SNX27^RNAi^ (Supplementary Fig. 4l–q), implying a distinct trafficking route for EGFR. To address whether the reduced apical localization may result from defects in protein synthesis or stability, we performed western blots with wing disc lysates. The protein levels of Ptp10D or EGFR did not change upon Vps35^RNAi^, Vps26^RNAi^ or Vps29^RNAi^ (Supplementary Fig. 5a–c), arguing that the observed defects are caused by aberrations in recycling to the apical plasma membrane. Taken together, this analysis suggests a bifurcation of EGFR and Ptp10D recycling in epithelial cells. As previously reported, Vps29 is a common component of the retromer and retriever complexes^43–45^. This prompted us to ask whether EGFR recycling is regulated by the retriever/CCC complex, which also associates with WASH. We first performed a mini-RNAi screen targeting conserved retriever and CCC subunits with *enGAL4* > GFP and analyzed adult viability, tissue defects or Ptp10D, aPKC and EGFR localization (Supplementary Table. 1). COMMD10, a conserved component of the CCC complex selectively assists retriever-dependent recycling^46,47^. The COMMD10^RNAi^ construct strongly affected EGFR but not Ptp10D accumulation in the P-compartment of wing discs (Supplementary Fig. 4i–v). Consistently, we also observed altered EGFR but not Ptp10D accumulations in the parts of wing discs expressing the retriever-specific SNX17^RNAi^ constructs (Supplementary Fig. 4g–s). Together, these results argue that EGFR and Ptp10D follow separate recycling routes. The WASH/retromer complex recycles Ptp10D and the WASH/retriever/CCC complex recycles EGFR.

**Fig. 4:**
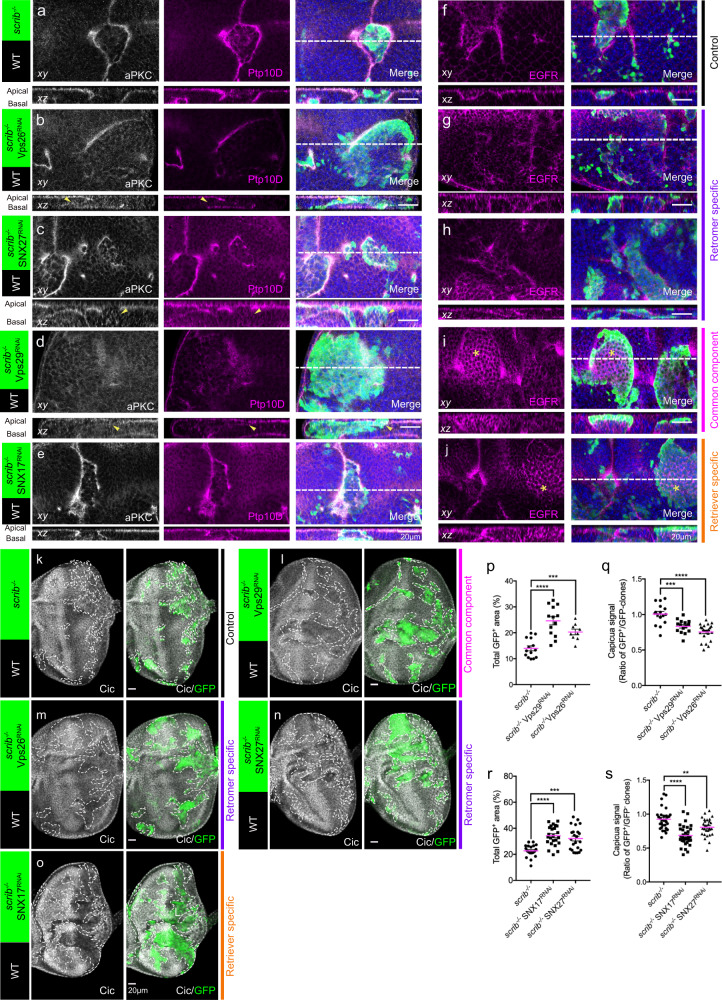
Ptp10D and EGFR recycling is facilitated by retromer and retriever complexes, respectively. **a**–**e** Immunochemistry analysis for aPKC and Ptp10D. Top images show xy confocal sections of eye disc bearing GFP-labelled *scrib*^−/−^ (**a**), *scrib*^−/−^ Vps26^RNAi^ (**b**), *scrib*^−/−^ SNX27^RNAi^ (**c**), *scrib*^−/−^ Vps29^RNAi^ (**d**) and *scrib*^−/−^ SNX17^RNAi^ (**e**) clones immunostained with anti-aPKC (gray), anti-Ptp10D (magenta), anti-GFP (green) and DAPI (blue); bottom images show *xz* cross sections. Dashed lines in the top right images with all the channels mark the positions ﻿of the cross-sections in the bottom images. Note that the interface Ptp10D and aPKC of *scrib*^−/−^ Vps26^RNAi^, *scrib*^−/−^ SNX27^RNAi^ and *scrib*^−/−^ Vps29^RNAi^ clones were disrupted (yellow arrowheads). **f–j** Immunochemistry analysis for EGFR. Top images show xy confocal sections of eye disc bearing GFP-labelled *scrib*^−/−^ (**f**), *scrib*^−/−^ Vps26^RNAi^ (**g**), *scrib*^−/−^ SNX27^RNAi^ (**h**), *scrib*^−/−^ Vps29^RNAi^ (**i**) and *scrib*^−/−−/−^ SNX17^RNAi^ (**j**) clones immunostained with anti-EGFR (magenta), anti-GFP (green) and DAPI (blue); bottom images show *xz* cross sections. Dashed lines in the top right images with all the channels mark the positions of the cross-sections in the bottom images. Note that EGFR increased inside the clones of *scrib*^−/−^ Vps29^RNAi^ and *scrib*^−/−^ SNX17^RNAi^ (yellow stars). **k–o** Eye-discs bearing GFP-labelled *scrib*^−/−^ (**k**), *scrib*^−/−^ Vps29^RNAi^ (**l**), *scrib*^−/−^ Vps26^RNAi^ (**m**), *scrib*^−/−^ SNX27^RNAi^ (**n**) and *scrib*^−/−^ SNX17^RNAi^ (**o**) clones immunostained with anti-Cic (gray) and anti-GFP (green). **p** Quantification of total GFP + area (%) in genotypes shown in *scrib*^−/−^ (*n* = 14, number of eye discs), *scrib*^−/−^ Vps29^RNAi^ (*n* = 13) and *scrib*^−/−^ Vps26^RNAi^ (*n* = 9). **q** Quantification of Capicua signal in *scrib*^−/−^ (*n* = 16, number of clones), *scrib*^−/−^ Vps29^RNAi^ (*n* = 15) and *scrib*^−/−^ Vps26^RNAi^ (*n* = 23) clones. **r** Quantification of total GFP^+^ area (%) in genotypes shown in *scrib*^−/−^ (*n* = 22, number of eye discs), *scrib*^−/−^ SNX17^RNAi^ (*n* = 24) and *scrib*^−/−^ SNX27^RNAi^ (*n* = 23). **s** Quantification of Capicua signal in *scrib*^−/−^ (*n* = 31, number of clones), *scrib*^−/−^ SNX17^RNAi^ (*n* = 32) and *scrib*^−/−^ SNX27^RNAi^ (*n* = 29) clones. p-s Data are mean ± s.e.m; **P < 0.005, ***P < 0.0005, ****P < 0.0001 by two tailed unpaired Mann-Whitney U-test. All scale bars are 20 μm.

To further dissect the impact of differential Ptp10D and EGFR recycling in tumor-suppressive cell competition, we selectively blocked retromer function with Vps26 ^RNAi^ or SNX27 ^RNAi^ in *scrib* mutant clones. In the boundary of *scrib*^−/−^ clones expressing either Vps26^RNAi^ or SNX27^RNAi^, Ptp10D, but not EGFR accumulation was severely disrupted (Fig. 4a–c, f–h), suggesting that retromer function is crucial for maintaining its interface localization. Inside these clones we detected an increase of EGFR levels. As expected, Vps26^RNAi^ or SNX27^RNAi^ clones also escaped elimination and showed reduced Cic levels compared to the *scrib*^−/−^ clones (Fig. 4p–s). This analysis indicates that Ptp10D is recycled to the *scrib*^−/−^ clone interface by the retromer, and interference with Ptp10D recycling route leads in EGFR signaling activation and overgrowth. Although EGFR accumulation was not detectably affected in the clone boundary, the increased levels of EGFR inside the retromer-deficient *scrib*^−/−^ clones suggests that retromer inactivation partially interferes EGFR recycling (Fig. 4g, h). As expected, in the *scrib*^−/−^ Vps29 ^RNAi^ clones, where both retromer and retriever were defective, all three cargoes were absent from the boundary and their levels were increased inside the clones (Fig. 4d, i). The *scrib*^−/−^ Vps29^RNAi^ clones also showed overgrowth and reduced Cic level (Fig. 4p, q). In the boundaries of *scrib*^−/−^ SNX17^RNAi^ clones, where the retriever complex is selectively compromised, Ptp10D and aPKC accumulated normally but EGFR was absent (Fig. 4e, j) and its levels were increased inside the clones (Fig. 4j). The *scrib*^−/−^ SNX17^RNAi^ clones also showed reduced Cic staining and overgrowth indicative of EGFR overactivation (Fig. 4r, s). In addition, knocking down another retriever-specific subunits Vps26C or CCC components COMMD3, COMMD10 and CCDC22 lead to the overgrowth of *scrib*^−/−^ clones, coupled with elevated EGFR signaling assessed by lowered Capicua levels (Supplementary Fig. 5i, j). Alternatively, to monitor EGFR activity, we performed immunostaining using an antibody against the principal EGFR effecter, di-phosphorylated Erk (dpERK)^48–50^. The dpERK staining in the wild-type eye-disc showed high intensity in morphogenic furrow (MF) in a row and clustered photoreceptor recruiting cells posterior to MF^48^ (Supplementary Fig. 5h). Consistently, ectopic dpERK was induced in the *scrib*^−/−^ clones expressing either retriever-specific RNAi (SNX17-RNAi and Vps26C-RNAi) and Vps29-RNAi ahead of morphogenic furrow but not in the *scrib*^−/−^ clones (Supplementary Fig. 5d–f). These data argue that the recycling routes of EGFR and Ptp10D/aPKC bifurcated in *scrib*^−/−^ clones. Our analysis of *scrib*^−/−^ Vps26^RNAi^ and *scrib*^−/−^SNX27^RNAi^ clones suggest that interference with retromer only partially affects EGFR targeting to the interface, but separates it from its inhibitor Ptp10D, consequently activating EGFR signaling and causing aberrant growth. On the other hand, inactivation of retriever function in *scrib*^−/−^ SNX17^RNAi^ strongly induced EGFR intracellular accumulation and signaling, while Ptp10D was targeted to the interface. This suggest that the increased EGFR signaling inside the clone is sufficient to induce overgrowth irrespective of the presence or absence of Ptp10D in the boundary.

## Discussion

**Fig. 5:**
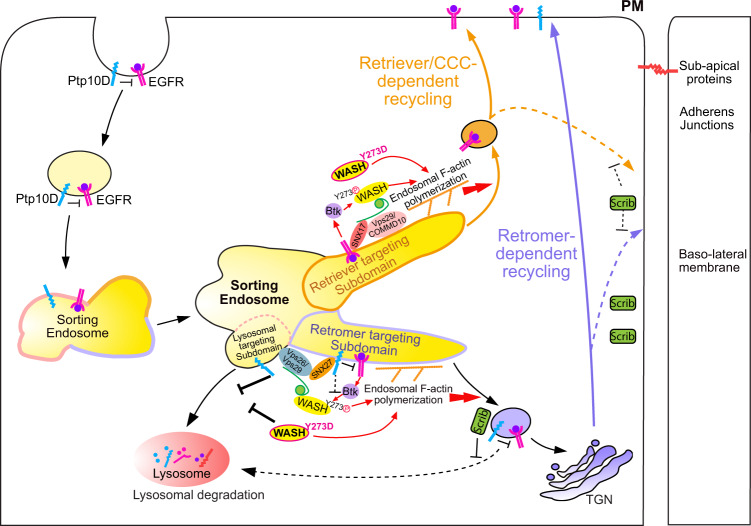
A proposed model for differential recycling routes of Ptp10D and EGFR in epithelial tissues. In the sorting endosomes, EGFR and Ptp10D located into different subdomains and follow bifurcated recycling routes. Ptp10D is recycled by the WASH/retromer complex while EGFR is predominantly recycled by the WASH/retriever/CCC complex. The efficiency of these recycling routes is regulated by endosomal F-actin polymerization boosted by phospho-WASH. Dysregulation of retromer or retriever-dependent recycling routes leads to separation of EGFR from its negative regulator Ptp10D, allowing EGFR signaling activation and aberrant clonal growth.

Overall, our genetic experiments in the *scrib*^−/−^ cell competition model reveal a tight and differential regulation of WASH activity in the retromer and the retriever recycling routes. Although we have not been able to directly detect WASH phosphorylation in the *scrib*^−/−^cell competition model, a phosphomimetic replacement of the conserved tyrosine (Y273 of WASH) induces both yki and EGFR activation, and clone overgrowth. Future work is needed to directly investigate the presence of phosphorylated WASH in imaginal disc clones. Several studies have shown that the cytoskeletal tension imbalances lead to yki activation, and tissue growth^33^. These studies mainly focused on cortical F-actin filaments polymerization regulated by capping proteins, Diaphanous (*Drosophila* formin) and cofilin^38,51–53^. Our work shows that excessive endosomal F-actin polymerization may interfere with the cortical cytoskeleton and triggers yki activation, suppression of apoptosis and *scrib*^−/−^ clone overgrowth. Proteomic analysis of retromer and retriever cargoes in human cell lines suggests that EGFR and PTPRJ (a human homologue of Ptp10D) are also recycled by different routes^44^. In our analysis of polarity-deficient epithelial cells, separation of EGFR from its negative regulator Ptp10D allowed increased EGFR signaling and subsequent tumorous growth. We speculate that in the subdomain of sorting endosome containing retriever cargos, EGFR may constantly stimulate Btk29A or other tyrosine kinases, which subsequently phosphorylate endogenous WASH. Phosphorylated WASH, boosts F-actin polymerization and presumably accelerates the basal level of retriever vesicles recycling directly to the plasma membrane. Conversely, in the sorting subdomain containing both retromer and retriever cargos, Ptp10D antagonizes EGFR or other RTKs. Thus, lower Btk29A activation leads to reduced-efficiency recycling by the retromer complex (Fig. 5). In all conditions interfering with retromer trafficking, including Ptp10D inactivation or WASH dysregulation we observed increased EGFR levels and activity inside the clones irrespective of its targeting to the plasma membranes raising the possibility that a small portion of EGFR may be recycled by the retromer or that retromer inactivation may interfere with retriever function. We do not exclude the possibility that other receptor tyrosine kinases together with/rather than EGFR regulate retromer function.

Recent work showed that the degree of overgrowth of *scrib*^−/−^ clones is influenced by diet composition, suggesting that systemic metabolic changes may interfere with cell competition in this context^3^. We note that although the average area of *scrib*^−/−^ Ptp10D^RNAi^ and *scrib*^−/−^ WASH^Y273D^ clones was consistently larger in all of our experiments compared to the area of *scrib*^−/−^ clones, we also detected an increased clone to clone variability upon Ptp10D inactivation or WASH^Y273D^ overexpression. Ptp10D and WASH regulate retromer trafficking, which also recycles nutrient transporters and other cargoes^54^ to the plasma membrane. The Ptp10D and WASH regulation of retromer transport may differentially affect growth in larvae with lower or higher nutrient access in crowded fly vials. A future challenge will be to further dissect out the mechanistic role of retromer recycling in *scrib*^−/−^ cell competition model. EGFR hyperactivation has been found in many epithelial cancers but EGFR-targeting therapies have limited effects in most solid tumors. The resistance to EGFR inhibitors treatment may be due to non-canonical EGFR signaling activation^55^. Previous studies reported that internalized EGFR continues signaling on the endosomes^56–58^. In agreement with these, we detected peripherally increased EGFR puncta and overgrowth of *scrib*^−/−^ clones upon inactivation of retriever components, suggesting that active EGFR is trapped and keeps signaling from the sorting endosomes. Although the physiological role of our model is not yet examined, our work provides insights into how WASH activation controls EGFR signaling and may help to devise alternative strategies for EGFR signaling inhibition.

## Methods

### *Drosophila* stocks and genetics

The following stocks are used: GFP labelled mitotic clones were induced in larval eye-antennal imaginal discs using the following strains:, y,w, eyFLP1;Act>y + >Gal4, UAS–GFP; FRT82B, Tub-Gal80 (82B tester-1), UAS-Dicer2; eyFLP5, Act>y + >Gal4, UAS-GFP; FRT82B, TubGal80 (82B tester-3), w Tub-Gal80, FRT19A; eyFLP5, Act>y + >Gal4, UAS- GFP (19 A tester) (kind gifts from Igaki’s lab). Additional strains used are as follows: *scrib*^1^ FRT82B (a kind gift from Rusten’s Lab), UAS-WASH^Y273D27^, UAS-Dicer2; *engrailed-gal4*::UAS-EGFP^25752^, *Nubbin-gal4* ^86106^, UAS-mCD8-GFP ^5137^, ex^697^ (ex-lacZ), UAS-bsk^DN 6409^, UAS-RasV12 ^4847^, UAS-EGFR^DN 5364^, UAS-Ptp10D-RNAi ^39001^, FRT82B^2035^ (Bloomington Drosophila Stock Center (BDSC)), UAS-WASH-RNAi^v39769^, UAS-Vps35-RNAi^v45570^, UAS-Vps29-RNAi^v101375^, UAS-Vps26- RNAi^v18396^, UAS-Arp2-RNAi^v101999^, UAS-Arp3-RNAi^v108951^ UAS-Btk29A-RNAi^v106962^, UAS-SNX27-RNAi^v108542^, UAS-SNX27-RNAi^v28457^, UAS-SNX17-RNAi^v109452^, UAS-SNX17-RNAi^v43798^, UAS-Vps26C-RNAi^v39758^, UAS-Vps26C-RNAi^v104859^, UAS-COMMD2-RNAi^v109443^, UAS-COMMD2-RNAi^v27894^, UAS-COMMD3-RNAi^v16400^, UAS-CCDC22-RNAi^v109399^, UAS-CCDC22-RNAi^v36172^, UAS-CCDC93-RNAi^v35267^, UAS-COMMD10-RNAi^v15483^, UAS-COMMD10-RNAi^v15482^ (Vienna Drosophila Research Center (VDRC)). All crosses were maintained at 25 °C.

### Immunochemistry

Third instar larvae were dissected in 1xPBS and fixed in 4% paraformaldehyde (PFA) for 20 min and stained. For dpERK staining, third instar larvae were dissected in cold 1xPBS and immediate fixed in 8% PFA for 20 min and followed with 10 min ice-cold ethanol treatment in −20 °C before further step. Samples were incubated with primary antibodies at the following dilutions: chicken anti-GFP (1:1000, Abcam, ab13970), rabbit anti-phospho-Histone H3 (Ser10) (1:200, Cell signaling, #9701), rabbit anti-Dcp1 (1:100, Cell signaling, #9578), mouse anti-WASH (1:5, Developmental Studies Hybridoma Bank(DSHB), P3H3) mouse anti-MMP1 (1:50, DSHB, cocktail 1:1:1 of 5H7B11, 3B8D12), mouse anti-Ptp10D (1:50, DSHB, cocktail 1:1 of 8B22F5 and 45E10), rabbit anti-aPKCζ (C-20) (1:250, Santa Cruz Biotechnology (SCBT), sc-216,), mouse anti-dEGFR (1:100, Sigma, E2906), guinea pig anti-capicua^41^ (1:1000, a kind gift from Edgar’s Lab), Alexa Fluor^TM^ Phalloidin 647 (1:50, Thermo Fisher, A22287), rabbit anti-ß-galactosidase (1:150, Cappel Laboratories, #0855976), rabbit anti-dpERK (1:200, cell signaling, #4370) guinea pig anti-DIAP1^59^ (1:200, a kind gift from Meier Pascal’s lab).

### Western blot analysis

10 wing discs were dissected from third instar larvae and homogenized in 10 μl of RIPA buffer and Protease inhibitor cocktail tablets (Roche, #11697498001). The lysates were centrifuged at maximum speed (30060 × *g*) for 10 min at 4 °C. The supernatant were further analyzed by SDS-PAGE gel. Blocking and antibody incubations were performed in TBST (TBS + 0.1% Tween 20). Samples were incubated with primary antibodies at the following dilutions: mouse anti-Ptp10D (1:50, DSHB, cocktail 1:1 of 8B22F5 and 45E10), mouse anti-dEGFR (1:200, sigma-Aldrich, E2906,) and rabbit anti-tubulin (1:1000, cell signaling, #2125). Three biological repeats were performed and calculated with Fiji.

### Quantification and statistical analysis

For the western blot quantification, the actual signal intensity of each band from the estimated molecular size was calculated after subtraction of the background, further normalized by the corresponding intensities of α-tubulin (as a loading control). Clone sizes were measured as relative ratio (%) of GFP-positive area versus total area of eye disc based on DAPI in 2D projections of image Z-stacks using Fiji Software. The capicua signal was measured as ratio of intensities in GFP + /GFP- clones using Fiji Software. Statistical analysis was performed with the Graphpad Prism 7 software. Data in the scatter plot graphs were expressed as mean ± s.e.m. Two-tailed unpaired t-test, either Mann-Whitney U test or Welch’s t test was used to estimate statistical significance. Statistical significance was denoted as follows: n.s *P* > 0.05, *P < 0.05, **P < 0.01, ***P < 0.001 and ****P < 0.0005. Exact *P* values were provided in the Source Data. Data were collected from at least three independent biological experiments.

### Reporting summary

Further information on research design is available in the Nature Research Reporting Summary linked to this article.

## Supplementary information

Supplementary Information

Reporting Summary

## Data Availability

All data supporting this study are available within the article, supplementary information, and source data. All reagents are available from the corresponding author upon request. Source data are provided with this paper.

## Source data

Source Data
