# Supplementary material for: WASH activation controls endosomal recycling and EGFR and Hippo signaling during tumor-suppressive cell competition
Source: Nat Commun. 2022 Oct 21;13:6243. doi: 10.1038/s41467-022-34067-1 (PMC9587002; doi:10.1038/s41467-022-34067-1)
Supplement: Supplementary file 1 — Supplementary Information [file 41467_2022_34067_MOESM1_ESM.pdf]

Supplementary Information

WASH activation controls endosomal recycling and EGFR and Hippo signaling during tumor-suppressive cell competition

Liu *et al*., 2022

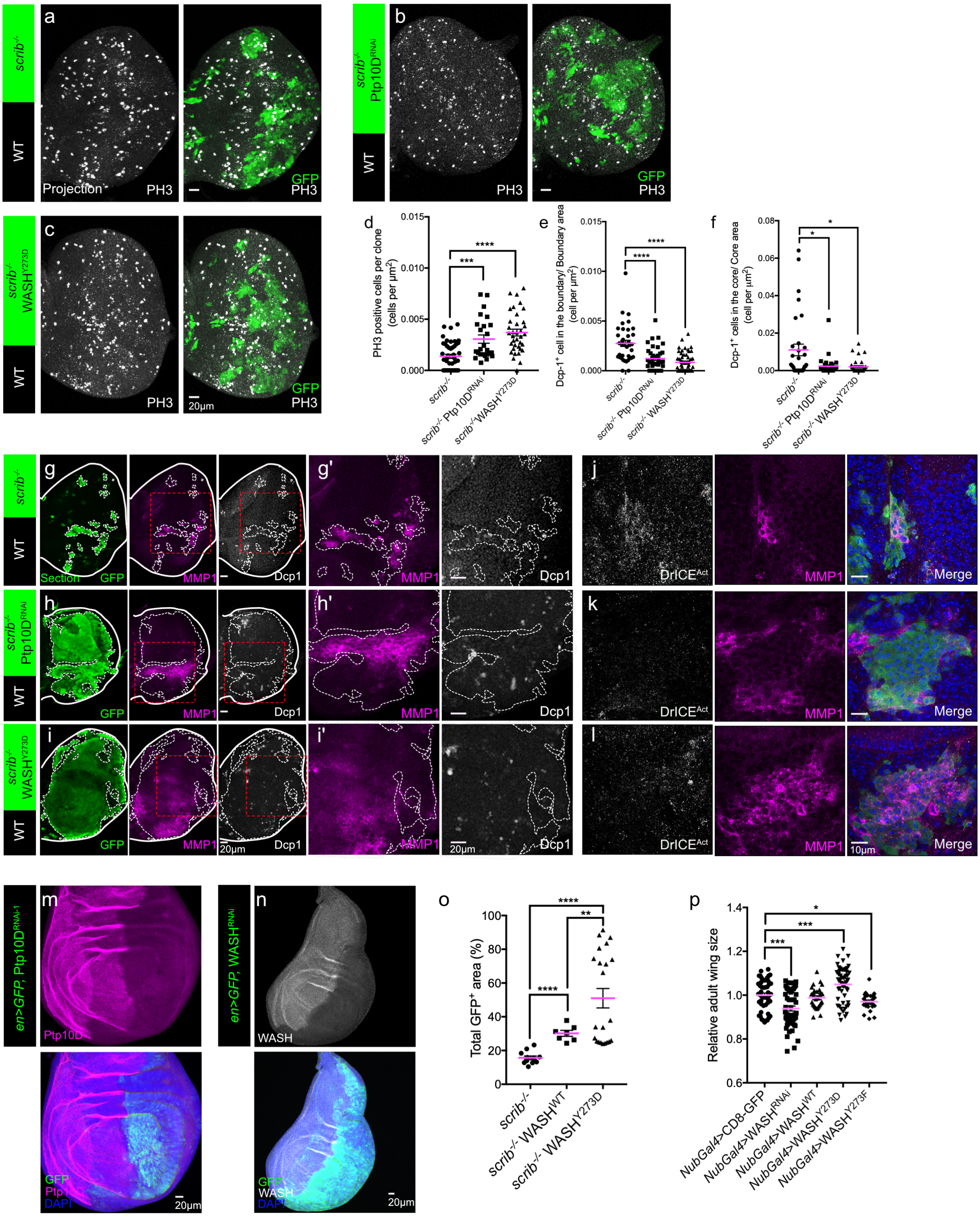

Figure S1

Supplementary Figure 1: WASH^{Y273D} overexpression increases proliferation, activates JNK pathway and inhibits apoptosis in *scrib* mutant clones.

(a-c) Eye-discs bearing GFP-labelled *scrib*$^{-/-}$ (a), *scrib*$^{-/-}$ Ptp10D^{RNAi} (b) or *scrib*$^{-/-}$ WASH^{Y273D} clones (c) immunostained with Phosphor-Histone 3 (PH3) (gray) and anti-GFP (green).

(d) Quantification of the number of PH3-positive cells per clone in *scrib*$^{-/-}$ (n = 46, number of clones), *scrib*$^{-/-}$ Ptp10D^{RNAi} (n = 25), and *scrib*$^{-/-}$ WASH^{Y273D} (n = 36) clones.

(e) Quantification of the number of Dcp-1 positive cells per clone boundary area in *scrib*$^{-/-}$ (n = 37, number of clones), *scrib*$^{-/-}$ Ptp10D^{RNAi} (n = 38), *scrib*$^{-/-}$ WASH^{Y273D} (n = 53) clones. (f) Quantification of the number of Dcp-1 positive cells per clone center area in *scrib*$^{-/-}$ (n = 32, number of clones), *scrib*$^{-/-}$ Ptp10D^{RNAi} (n = 34), *scrib*$^{-/-}$ WASH^{Y273D} (n = 35) clones.

(g-i) Eye-discs bearing GFP-labelled *scrib*$^{-/-}$ (G), *scrib*$^{-/-}$ Ptp10D^{RNAi} (H) or *scrib*$^{-/-}$ WASH^{Y273D} clones (I) immunostained with anti-GFP (green) , anti-MMP1(magenta), Dcp-1 (gray) and DAPI (blue). Dashed lines show GFP-labeled clone area.

(g'-i') show magnified images of (g-i) with anti-MMP1 and anti-Dcp1 staining. Scale bars, 20μm. (j-l) Eye-discs bearing GFP-labelled *scrib*$^{-/-}$ (j), *scrib*$^{-/-}$ Ptp10D^{RNAi} (k) or *scrib*$^{-/-}$ WASH^{Y273D} clones (l) immunostained with anti-GFP (green) , anti-MMP1(magenta), anti-cleaved *Drosophila* ICE (Asp230) (DrICE^{act}) (gray) and DAPI (blue).

(m) Wing-discs of *engrailed-GAL4*::UAS-GFP, UAS-Ptp10D^{RNAi} shown as confocal section, immunostained with anti-Ptp10D (magenta), anti-GFP (green) and DAPI (blue). (n) Wing-discs of *engrailed-GAL4*::UAS-GFP, UAS-WASH^{RNAi} shown as confocal section, immunostained with anti-WASH (gray), anti-GFP (green) and DAPI (blue).

(o) Quantification of total GFP^{+} area (%) of *scrib*$^{-/-}$ (n = 12), *scrib*$^{-/-}$ WASH^{WT} (n = 7) and *scrib*$^{-/-}$WASH^{Y273D} (n = 22). (*scrib*$^{-/-}$, *scrib*$^{-/-}$ WASH^{Y273D} data reproduced from Fig. 1j for comparison).

(p) Relative wing sizes in adult female flies of *Nubbin-Gal4*>CD8-GFP (control, n = 52 adult wings), *Nubbin-Gal4*>WASH^{RNAi} (n = 56), *Nubbin-Gal4*>WASH^{WT} (n = 29), *Nubbin-Gal4*>WASH^{Y273D} (n = 54) and *Nubbin-Gal4*>WASH^{Y273F} (n = 26).

Data (d-f, p) are mean ± s.e.m; * $P < 0.05$, ** $P < 0.005$, *** $P < 0.001$, by Mann-Whitney U-test. Data (o) are mean ± s.e.m ; ** $P < 0.005$, **** $P < 0.0001$ by Welch's t test.

Scale bars (j-k), 10μm. All other scale bars, 20μm.

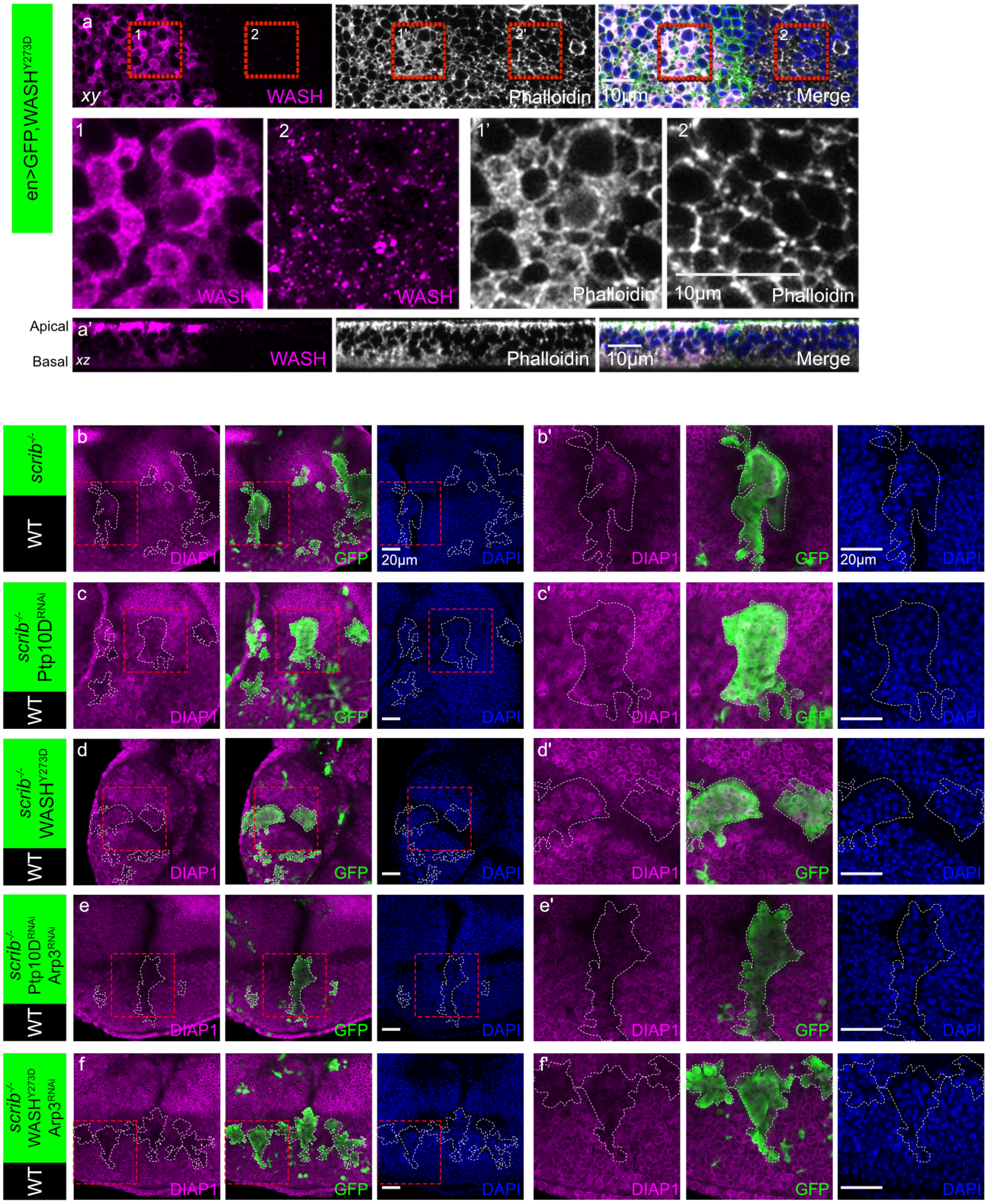
en>GFP,WASH Y273D
a
xy
WASH
Phalloidin
10μm
Merge
1
2
1'
2'
Apical
Basal
a'
xz
scrib-/-
WT
b
DIAP1
GFP
20μm
DAPI
b'
scrib-/- Ptp10D RNAi
c
c'
scrib-/- WASH Y273D
d
d'
scrib-/- Ptp10D RNAi Arp3 RNAi
e
e'
scrib-/- WASH Y273D Arp3 RNAi
f
f'

Figure S2

Supplementary Figure 2: Phospho-WASH induces F-actin polymerization in wing disc epithelium.

(a) Wing-discs of *engrailed-GAL4*::UAS-GFP, UAS-WASH^{Y273D} shown as sections (a) or optical cross-sections (a'), immunostained with phalloidin (gray), anti-WASH (magenta), anti-GFP (green) and DAPI (blue). Lower rows in 1,1' (cells overexpressing WASH^{Y273D}) and 2,2' (wild-type cells, internal control) depict zoomed view of areas denoted by the rectangular frames. Note that phalloidin increased in cytoplasmic region upon WASH^{Y273D}overexpression. Scale bars, 10µm.

(b-f') Eye-discs bearing GFP-labelled *scrib*$^{-/-}$ (b), *scrib*$^{-/-}$ Ptp10D^{RNAi} (c), *scrib*$^{-/-}$ WASH^{Y273D} (d) *scrib*$^{-/-}$ Ptp10D^{RNAi} Arp3^{RNAi} (e) and *scrib*$^{-/-}$ WASH^{Y273D} Arp3^{RNAi} (f) clones immunostained with anti-DIAP1 (magenta), anti-GFP (green) and DAPI (blue). (b') (c') (d') (e') (f') show magnified images of (b) (c) (d) (e) (f).

Scale bars, 20µm.

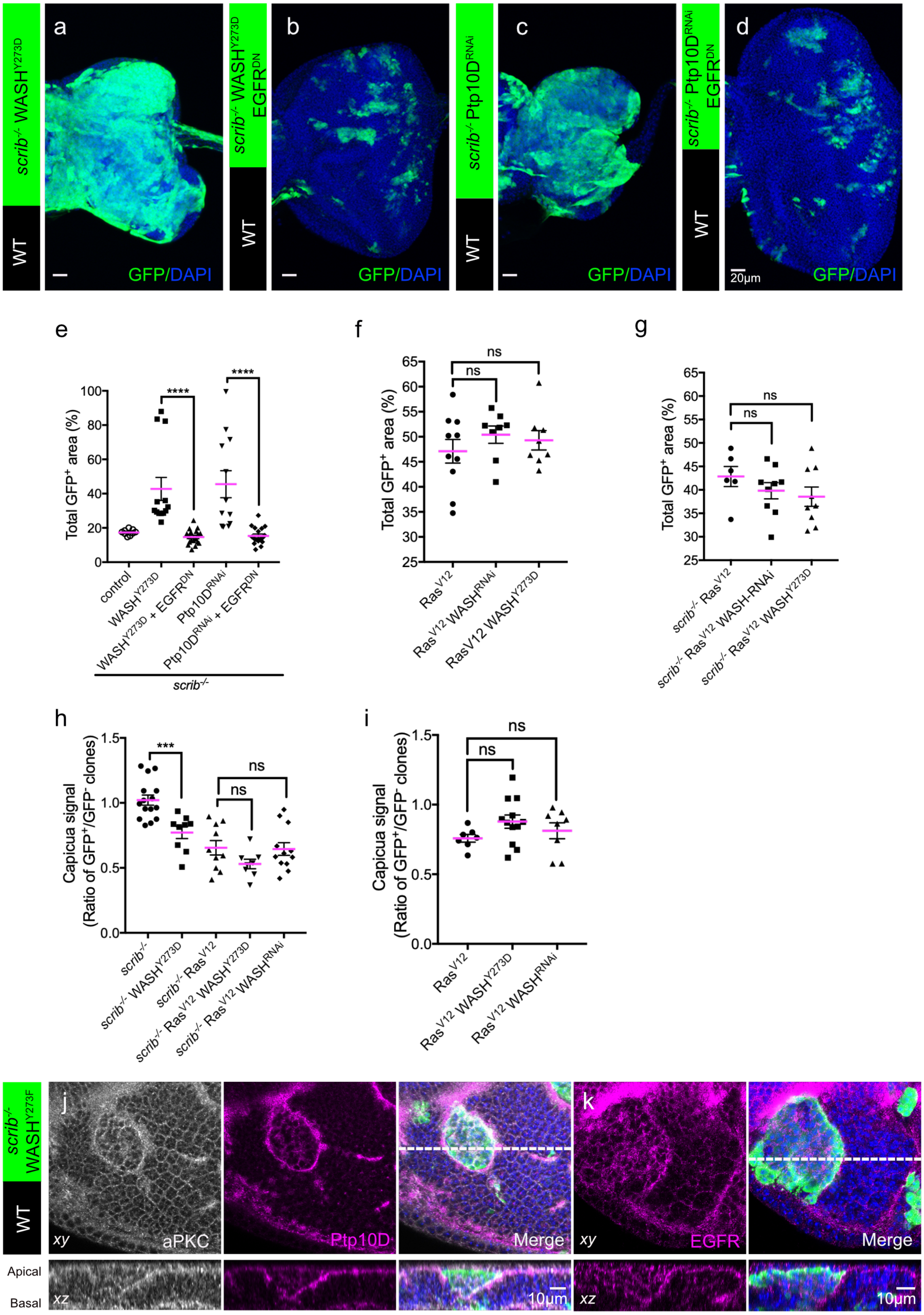

a
b
c
d
scrib-/- WASHY273D
scrib-/- WASHY273D EGFRDN
scrib-/- Ptp10DRNAi
scrib-/- Ptp10DRNAi EGFRDN
WT
GFP/DAPI
20µm
e
f
g
h
i
Total GFP+ area (%)
control
WASHY273D
WASHY273D + EGFRDN
Ptp10DRNAi
Ptp10DRNAi + EGFRDN
scrib-/-
****
ns
RasV12
RasV12 WASHRNAi
RasV12 WASHY273D
scrib-/- RasV12
scrib-/- RasV12 WASH-RNAi
scrib-/- RasV12 WASHY273D
Capicua signal
(Ratio of GFP+/GFP- clones)
***
scrib-/-
scrib-/- WASHY273D
scrib-/- RasV12 WASHRNAi
j
k
scrib-/- WASHY273F
xy
xz
aPKC
Ptp10D
Merge
EGFR
Apical
Basal
10µm

Figure S3

Supplementary Figure 3: WASH^{Y273D} regulates EGFR signaling upstream of Ras in the *scrib* mutant clones.

(a-d) Eye-discs bearing GFP-labelled *scrib*$^{-/-}$ WASH^{Y273D} (a), *scrib*$^{-/-}$ WASH^{Y273D} EGFR^{DN} (b), *scrib*$^{-/-}$ Ptp10D^{RNAi} (c) and *scrib*$^{-/-}$ Ptp10D^{RNAi} EGFR^{DN} (d) clones immunostained with anti-GFP (green) and DAPI (blue). Scale bars, 20μm.

(e) Quantification for total GFP^{+} area (%) of *scrib*$^{-/-}$ (n = 9, number of eye discs), *scrib*$^{-/-}$ WASH^{Y273D} (n = 13), *scrib*$^{-/-}$ WASH^{Y273D} EGFR^{DN} (n = 18), *scrib*$^{-/-}$ Ptp10D^{RNAi} (n = 12) and *scrib*$^{-/-}$ Ptp10D^{RNAi} EGFR^{DN} (n = 20) clones. (*scrib*$^{-/-}$, *scrib*$^{-/-}$ WASH^{Y273D} and *scrib*$^{-/-}$ Ptp10D^{RNAi} data reproduced from Figure 2F for comparison. The experiment for Figure 2F and Figure S3E was performed at the same time).

(f) Quantification for total GFP^{+} area (%) of Ras^{V12} (n = 6, number of eye discs), Ras^{V12} WASH^{RNAi} (n = 9), Ras^{V12} WASH^{Y273D} (n = 9).

(g) Quantification for total GFP^{+} area (%) of *scrib*$^{-/-}$ Ras^{V12} (n = 10, number of eye discs), *scrib*$^{-/-}$ Ras^{V12} WASH^{RNAi} (n = 8), *scrib*$^{-/-}$ Ras^{V12} WASH^{Y273D} (n = 8).

(h) Quantification for Capicua (GFP-positive / negative clones ratio) in the *scrib*$^{-/-}$ Ras^{V12} (n = 10, number of clones), *scrib*$^{-/-}$ Ras^{V12} WASH^{Y273D} clones (n = 8) and *scrib*$^{-/-}$ Ras^{V12} WASH^{RNAi} clones (n = 12).

(i) Quantification for Capicua (GFP-positive / negative clones ratio) in the Ras ^{V12} clones (n = 7, number of clones), Ras^{V12} WASH^{Y273D} clones (n = 12) and Ras^{V12} WASH^{RNAi} clones (n = 8). e-i Data are mean ± s.e.m ; **** $P < 0.0001$, n.s > 0.05 by two tailed unpaired Mann-Whitney U-test.

(j,k) Immunochemistry analysis for Ptp10D and aPKC (j) or EGFR (k). Top images show xy confocal sections of eye disc bearing GFP-labelled *scrib*$^{-/-}$ WASH^{Y273F} clones immunostained with anti-Ptp10D (gray), anti-aPKC (magenta), anti-GFP (green) and DAPI (blue) or immunostained with anti-EGFR (magenta), anti-GFP (green) and DAPI (blue); bottom images show xz cross sections. Dashed lines in the top right images with all the channels mark the positions of the cross-sections in the bottom images. Scale bars, 10μm.

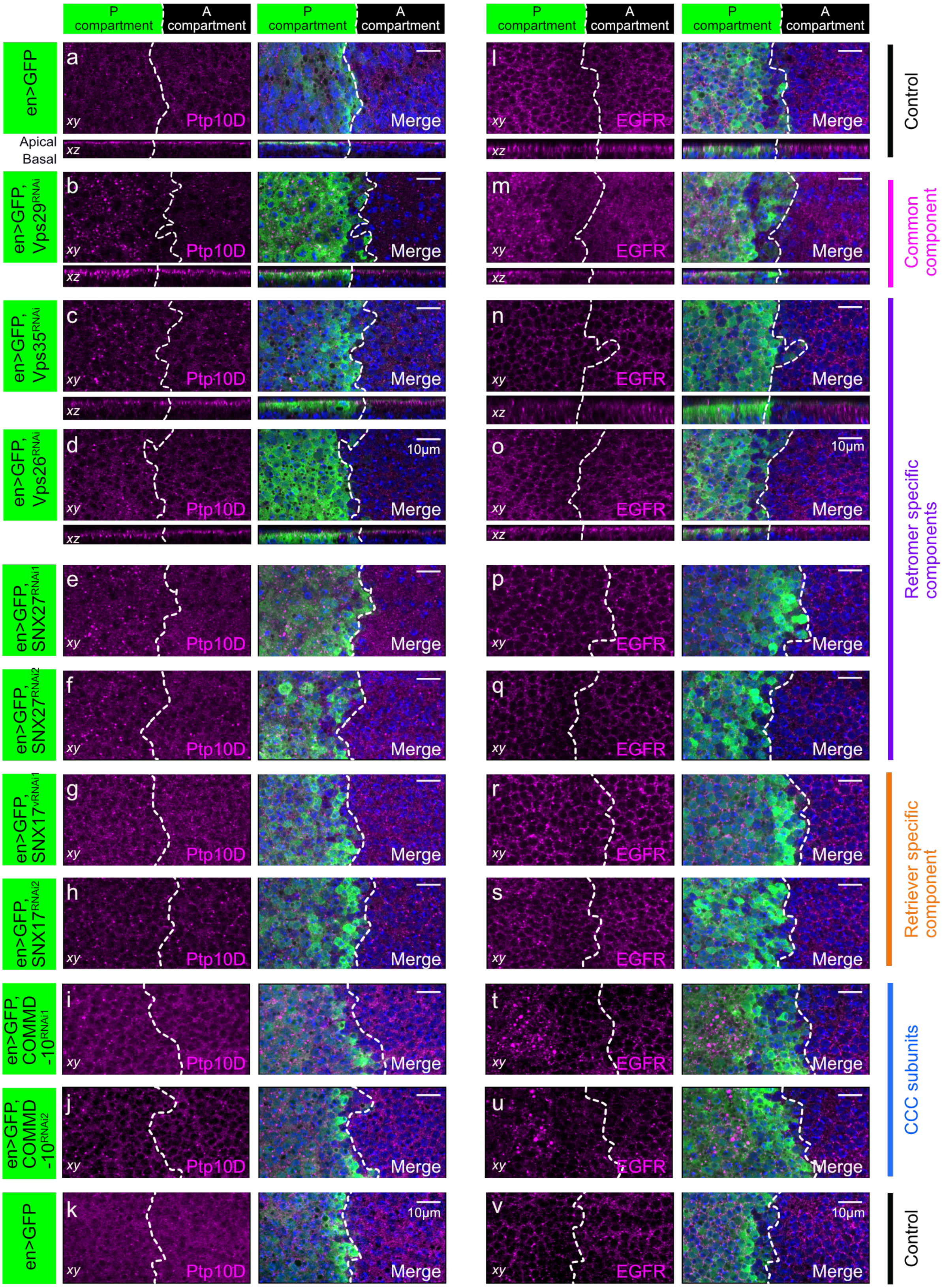
P compartment
A compartment
en>GFP
Apical
Basal
en>GFP, Vps29RNAi
en>GFP, Vps35RNAi
en>GFP, Vps26RNAi
en>GFP, SNX27RNAi1
en>GFP, SNX27RNAi2
en>GFP, SNX17vRNAi1
en>GFP, SNX17RNAi2
en>GFP, COMMD-10RNAi1
en>GFP, COMMD-10RNAi2
en>GFP
Ptp10D
EGFR
Merge
xy
xz
10μm
Control
Common component
Retromer specific components
Retriever specific component
CCC subunits
Control

Figure S4

Supplementary Figure 4: Ptp10D and EGFR localization are regulated by retromer complex and retriever complex/CCC complex, respectively in the imaginal wing discs.
(a-k) Airyscan confocal sections (top images) and orthogonal view (bottom images) showing wing-discs with *engrailed-Gal4::*UAS-GFP (a, k) (control) or *engrailed-Gal4* UAS-GFP driving Vps29^{RNAi} (b), Vps35^{RNAi} (c), Vps26^{RNAi} (d), SNX27^{RNAi-1}(e), SNX27^{RNAi-2}(f), SNX17^{RNAi-1}(g), SNX17^{RNAi-2} (h), COMMD10^{RNAi-1} (i), and COMMD10^{RNAi-2} (j) immunostained with anti-Ptp10D (magenta), anti-GFP (green) and DAPI (blue). Dashed lines present the boundary of posterior compartment (P-compartment, expressing GFP) and anterior compartment (A-compartment) defined by *engrailed-GAL4*::UAS-GFP. Note that apical localized Ptp10D is defective upon knock down of retromer components, Vps29, Vps35 and Vps26. Scale bars, 10µm.
(l-v) Airyscan confocal sections (top images) and orthogonal view (bottom images) showing wing-discs with *engrailed-Gal4,*UAS-GFP (l,v) (control) or *engrailed-Gal4* UAS-GFP driving Vps29^{RNAi} (m), Vps35^{RNAi} (n), Vps26^{RNAi} (o), SNX27^{RNAi-1}(p), SNX27^{RNAi-2}(q), SNX17^{RNAi-1}(r), SNX17^{RNAi-2} (s), COMMD10^{RNAi-1} (t), and COMMD10^{RNAi-2} (u) immunostained with anti-EGFR (magenta), anti-GFP (green) and DAPI (blue). Dashed lines present the boundary of P-compartment and A-compartment defined by *engrailed-GAL4* UAS-GFP. Note that EGFR is defective upon knock down of Vps29 (common component) but not Vps35 and Vps26 (retromer specific). EGFR puncta (t,u) dramatically increased upon COMMD10 knock-down. Scale bars, 10µm.

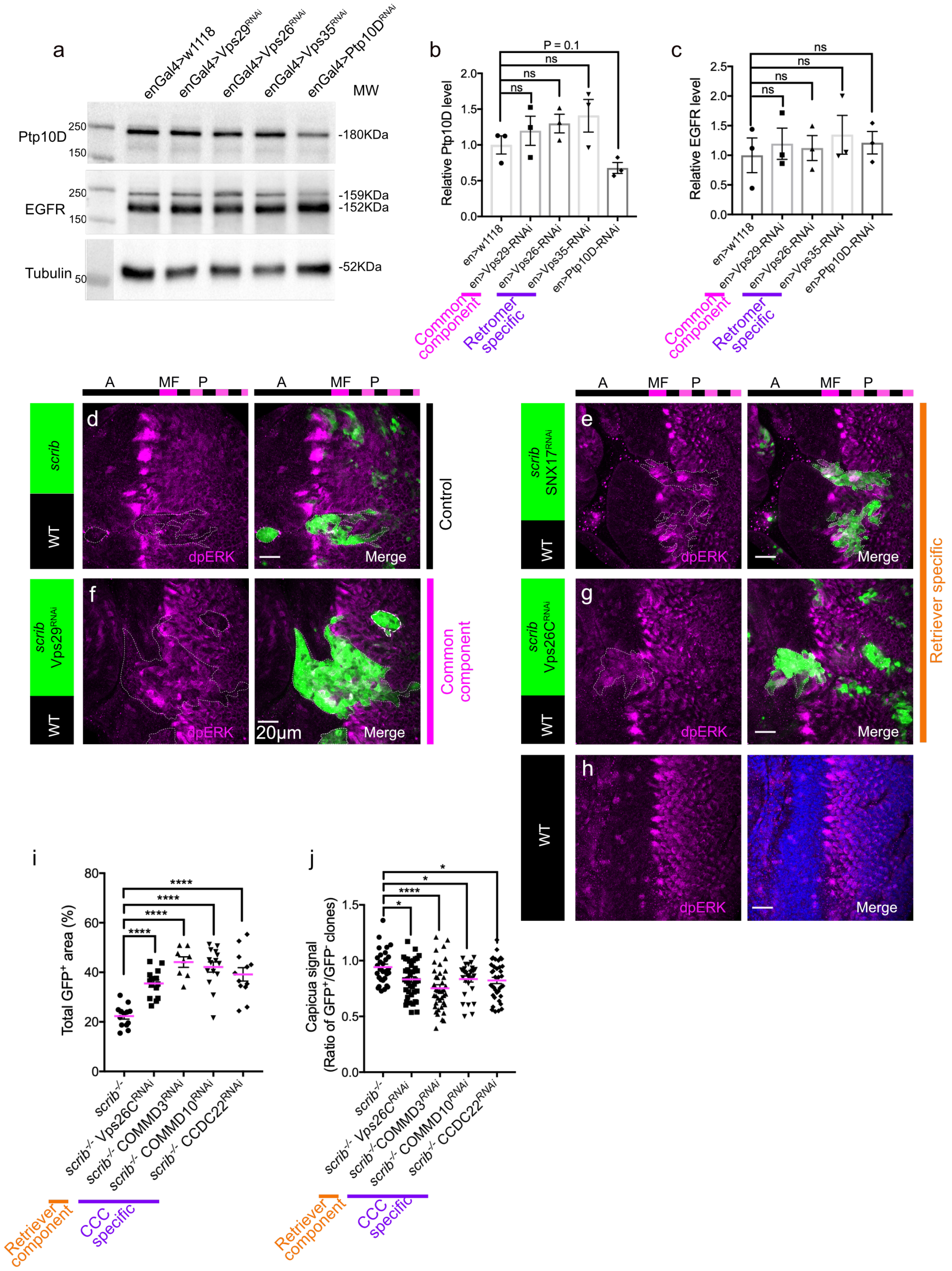

Figure S5

Supplementary Figure 5: Protein levels of Ptp10D and EGFR are not affected by inactivation of retromer subunits or common component Vps29.

(a) Western blot analysis showing the expression levels of Ptp10D and EGFR in wing-discs lysates of *engrailed-Gal4::*UAS-GFP (control) or *engrailed-Gal4::*UAS-GFP driving $Vps29^{RNAi}$, $Vps26^{RNAi}$, $Vps35^{RNAi}$, $Ptp10D^{RNAi}$. Anti-tubulin used as loading control. Each lane contains 10 wing-discs.

(b,c) Plot depicting the relative Ptp10D/a-tubulin signals (b) and EGFR/a-tubulin signals (c), normalized to control. The western blot band intensities were measured from 3 independent experiments. Note that there is no significant change in Ptp10D or EGFR protein level. Data are mean ± s.e.m. n.s P= 0.7 by two tailed unpaired Mann-Whitney U-test.

(d-h) Eye-discs bearing GFP-labelled *scrib*$^{-/-}$ (d), *scrib*$^{-/-}$ $SNX17^{RNAi}$ (e), *scrib*$^{-/-}$ $Vps29^{RNAi}$ (f) *scrib*$^{-/-}$ $Vps26C^{RNAi}$ (g) and eye-discs of w^{1118} (h) immunostained with anti-dpERK (magenta), anti-GFP (green) and DAPI (blue). A, anterior to MF; MF, morphogenic furrow; P, posterior to MF. Scale bars, 20 µm.

(i) Quantification of total GFP^{+} area (%) of *scrib*$^{-/-}$ (n = 13, number of eye-discs), *scrib*$^{-/-}$ $Vps26C^{RNAi}$ (n = 14), *scrib*$^{-/-}$ $COMMD3^{RNAi}$ (n = 8) and *scrib*$^{-/-}$ $COMMD10^{RNAi}$ (n = 15) and *scrib*$^{-/-}$ $CCDC22^{RNAi}$ (n = 12) clones.

(j) Quantification of Capicua signal in *scrib*$^{-/-}$ (n = 30, number of clones), *scrib*$^{-/-}$ $Vps26C^{RNAi}$ (n = 43), *scrib*$^{-/-}$ $COMMD3^{RNAi}$ (n = 40) and *scrib*$^{-/-}$ $COMMD10^{RNAi}$ (n = 29) and *scrib*$^{-/-}$ $CCDC22^{RNAi}$ (n = 38) clones. i, j Data are mean ± s.e.m; * $P < 0.05$, **** $P < 0.0001$ by two tailed unpaired Mann-Whitney U-test.

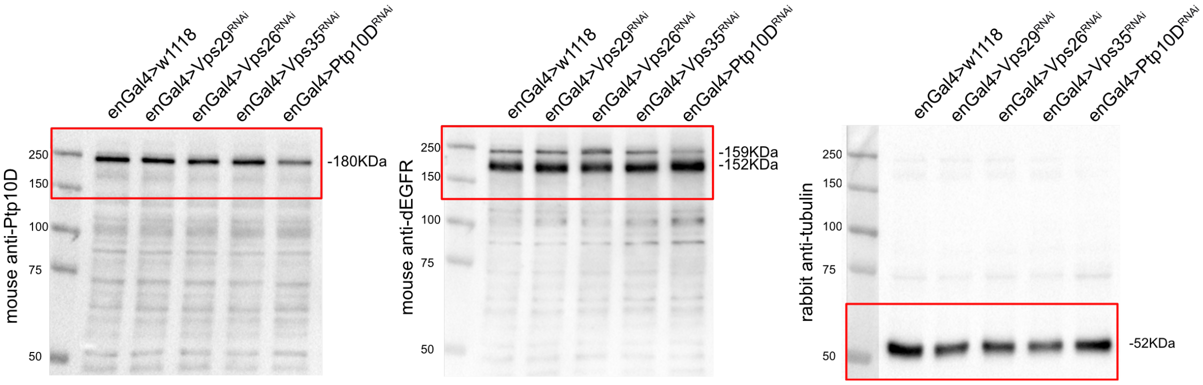

Supplementary Figure 6: Unprocessed Western blots.
Uncropped original blots used in the Supplementary figure 5a. Red boxes indicate the region presented in the figure.

Supplementary Table 1: Summary of mini-RNAi screen

	Genotypes	VDRC lines	Adult wing phenotype	Ptp10D Staining	EGFR staining
Retriever Specific	*enGal4*>Vps26C^{RNAi}	104859	N.S	N.S	N.S
		39758	Smaller P compartment and vein defect.	N.S	Less
	enGal4>SNX17^{RNAi}	109452	N.S (Smaller wings with *Nubbin*-GAL4)	N.S	N.S
		43798	N.S	N.S	N.S
	enGal4>Vps35L^{RNAi}	35408	N.S	N.T	N.T
		105632	N.S	N.T	N.T
CCC Specific	*enGal4*>COMMD2^{RNAi}	109443	Smaller P compartment	N.S	N.S
		27894	N.S	N.S	N.S
	enGal4>COMMD3^{RNAi}	16400	Smaller P compartment	N.S	Less
	enGal4>COMMD10^{RNAi}	15482	Shrinked wings, very few survived adults	Small intracellular puncta	Big intracellular puncta
		15483	Shrinked wings, few survived adults	Small intracellular puncta	Big intracellular puncta
	enGal4>CCDC22^{RNAi}	109399	N.S	N.S	N.S
		36172	N.S	N.S	N.S
	enGal4>CCDC93^{RNAi}	35267	Embryonic lethal	N.S	N.S

P compartment, posterior region; N.S, No significant change; N.T, Not tested.
