# Supplementary material for: WASH activation controls endosomal recycling and EGFR and Hippo signaling during tumor-suppressive cell competition
Source: Nat Commun. 2022 Oct 21;13:6243. doi: 10.1038/s41467-022-34067-1 (PMC9587002; doi:10.1038/s41467-022-34067-1)
Supplement: Supplementary file 2 — Reporting Summary [file 41467_2022_34067_MOESM2_ESM.pdf]

nature portfolio

Corresponding author(s): Christos Samakovlis

Last updated by author(s): Oct 3, 2022

Reporting Summary

Nature Portfolio wishes to improve the reproducibility of the work that we publish. This form provides structure for consistency and transparency in reporting. For further information on Nature Portfolio policies, see our Editorial Policies and the Editorial Policy Checklist.

Statistics

For all statistical analyses, confirm that the following items are present in the figure legend, table legend, main text, or Methods section.

n/a	Confirmed	
☐	☒	The exact sample size (n) for each experimental group/condition, given as a discrete number and unit of measurement
☐	☒	A statement on whether measurements were taken from distinct samples or whether the same sample was measured repeatedly
☐	☒	The statistical test(s) used AND whether they are one- or two-sided<br>*Only common tests should be described solely by name; describe more complex techniques in the Methods section.*
☒	☐	A description of all covariates tested
☐	☒	A description of any assumptions or corrections, such as tests of normality and adjustment for multiple comparisons
☐	☒	A full description of the statistical parameters including central tendency (e.g. means) or other basic estimates (e.g. regression coefficient) AND variation (e.g. standard deviation) or associated estimates of uncertainty (e.g. confidence intervals)
☐	☒	For null hypothesis testing, the test statistic (e.g. F, t, r) with confidence intervals, effect sizes, degrees of freedom and P value noted<br>*Give P values as exact values whenever suitable.*
☒	☐	For Bayesian analysis, information on the choice of priors and Markov chain Monte Carlo settings
☒	☐	For hierarchical and complex designs, identification of the appropriate level for tests and full reporting of outcomes
☒	☐	Estimates of effect sizes (e.g. Cohen's d, Pearson's r), indicating how they were calculated

Our web collection on statistics for biologists contains articles on many of the points above.

Software and code

Policy information about availability of computer code

Data collection	Image acquisition was performed using confocal microscope ZEISS LSM780 or ZEISS LSM800 confocal microscopes with ZEN software (ZEN Blue v2.1).
Data analysis	Images were analyzed and processed using Fiji2 (version 1.51h). Graphpad Prism 7 was used to analyze and graph the data.

For manuscripts utilizing custom algorithms or software that are central to the research but not yet described in published literature, software must be made available to editors and reviewers. We strongly encourage code deposition in a community repository (e.g. GitHub). See the Nature Portfolio guidelines for submitting code & software for further information.

Data

Policy information about availability of data

All manuscripts must include a data availability statement. This statement should provide the following information, where applicable:

- Accession codes, unique identifiers, or web links for publicly available datasets
- A description of any restrictions on data availability
- For clinical datasets or third party data, please ensure that the statement adheres to our policy

The data that support this study are available from the corresponding author upon reasonable request.

Field-specific reporting

Please select the one below that is the best fit for your research. If you are not sure, read the appropriate sections before making your selection.

☒ Life sciences ☐ Behavioural & social sciences ☐ Ecological, evolutionary & environmental sciences

For a reference copy of the document with all sections, see nature.com/documents/nr-reporting-summary-flat.pdf

Life sciences study design

All studies must disclose on these points even when the disclosure is negative.

Sample size	No sample size calculation was performed in advance. Sample size sufficiency was based on previous studies in the same field (Wang et al., Nature Cell Biology. 2009). Sample sizes of of 5-10 discs are enough for revealing the phenotype. All sample sizes were indicated in each figure legend. Sample sizes (n) either define the number of imaginal discs or the number of clones as noted in the related figure legends.
Data exclusions	No data were excluded from the analysis.
Replication	Experiments in this work were replicated through repeated testing on individual larvae. Sample sizes were presented clearly in the figure legends. To knock-down with RNAi or overexpress transgenes in the wing discs or generate MARCM clone in the eye-antennal discs, all the experiments required crosses and we used the progenies from the crosses. We dissected and analyzed wandering 3rd instar larvae with correct genotypes from 3-5 independent population of progenies (individual fly vials) per sample for testing reproducibility. We did not notice any limitations in reproducibility.
Randomization	The experiments were not randomized. All samples were allocated based on genotypes and developmental stage (wandering 3rd instar larvae). Appropriate control groups were assayed in parallel.
Blinding	The blinding was not possible when picking up the correct genotypes. The correct genotypes contain GFP clones in the eye discs of 3rd instar wandering larvae, selected by chromosomal balancers tagged with fluorescence. Within the correct genotypic group, we performed blinding analysis.

Reporting for specific materials, systems and methods

We require information from authors about some types of materials, experimental systems and methods used in many studies. Here, indicate whether each material, system or method listed is relevant to your study. If you are not sure if a list item applies to your research, read the appropriate section before selecting a response.

Materials & experimental systems

n/a	Involved in the study
☐	☒ Antibodies
☒	☐ Eukaryotic cell lines
☒	☐ Palaeontology and archaeology
☐	☒ Animals and other organisms
☒	☐ Human research participants
☒	☐ Clinical data
☒	☐ Dual use research of concern

Methods

n/a	Involved in the study
☒	☐ ChIP-seq
☒	☐ Flow cytometry
☒	☐ MRI-based neuroimaging

Antibodies

Antibodies used

For immunostaining, the following primary antibodies and dilutions were used:
chicken anti-GFP (1:1000, Abcam, ab13970),
rabbit anti-phospho-Histone H3 (Ser10) (1:200, Cell signaling, #9701),
rabbit anti-Dcp1 (1:100, Cell signaling, #9578),
rabbit anti-Cleaved Drosophila ICE (drICE) (Asp230) (1:100,Cell signaling, #9478)
mouse anti-WASH (1:5, Developmental Studies Hybridoma Bank(DSHB), P3H3),
mouse anti-MMP1 (1:50, DSHB, cocktail 1:1:1 of 5H7B11, 3B8D12, 3A6B4),
mouse anti-Ptp10D (1:50, DSHB, cocktail 1:1 of 8B22F5 and 45E10),
rabbit anti-aPKCζ (C-20) (1:250, Santa Cruz Biotechnology (SCBT), sc-216,)
mouse anti-dEGFR (1:100, Sigma, E2906),
guinea pig anti-capicua (1:800, a kind gift from Edgar' Lab, Ref41. Jin Y et al., 2015. Plos Genetics),
Alexa FluorTM Phalloidin 647 (1:50, Thermo Fisher, A22287),
rabbit anti-ß-galactosidase (1:150, Cappel Laboratories, #0855976).
anti-dpERK (1:200, cell signaling, #4370)
guinea pig anti-DIAP1 (1:200, a kind gift from Meier Pascal's lab, Ref59. Wilson R et al.,2002. Nature cell Biology).

For western blot analysis, the following primary antibodies and dilutions were used:
mouse anti-Ptp10D (1:50, DSHB, cocktail 1:1 of 8B22F5 and 45E10),
mouse anti-dEGFR (1:200, sigma-Aldrich, E2906)
rabbit anti-tubulin (1:1000, cell signaling, #2125).

Validation

All the commercial antibodies were validated by the suppliers. GP anti-DIAP1 and GP anti-capicua were validated in previous publications (Reference 41 and 59).

Animals and other organisms

Policy information about studies involving animals; ARRIVE guidelines recommended for reporting animal research

Laboratory animals

The stock numbers and the origin of the fly strains used in the study were described in the material and methods. We used Drosophila melanogaster third instar larvae, of the indicated genotypes, at wandering stage. Group of male and female larvae were used for all experiments. The sex determination was not considered in this study.

Wild animals

This study did not involve wild animals.

Field-collected samples

This study did not involve samples from the field.

Ethics oversight

The use of Drosophila does not require ethical approval.

Note that full information on the approval of the study protocol must also be provided in the manuscript.
